# 1,5-Anhydro-3,6-di-*O*-benzyl-2-de­oxy-1,2-*C*-dichloro­methyl­ene-d-*glycero*-d-*gulo*-hexitol

**DOI:** 10.1107/S1600536811040815

**Published:** 2011-10-08

**Authors:** Henok H. Kinfe, Blessing A. Aderibigbe, Alfred Muller

**Affiliations:** a Research Center for Synthesis and Catalysis, Department of Chemistry, University of Johannesburg (APK Campus), PO Box 524, Auckland Park, Johannesburg 2006, South Africa

## Abstract

In the title compound, C_21_H_22_Cl_2_O_4_, the pyranosyl ring adopts a twist-boat conformation with the *O*-benzyl groups in equatorial positions. In the crystal, O—H⋯O hydrogen bonding results in infinite chains of mol­ecules along [100]. The structure is further consolidated by weak C—H⋯O, C—H⋯Cl and C—H⋯π inter­actions. The absolute structure was determined.

## Related literature

For *O*-benzyl deprotection methodologies, see: Akiyama *et al.* (1991 ▶). For a related structure, see: Shanmugasundaram *et al.* (2002 ▶). For ring puckering analysis, see: Cremer & Pople (1975 ▶).
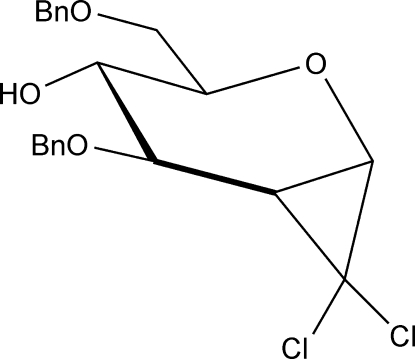


## Experimental

### 

#### Crystal data

C_21_H_22_Cl_2_O_4_
*M* *_r_* = 409.29
Orthorhombic, 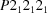

*a* = 5.2985 (1) Å
*b* = 18.8511 (3) Å
*c* = 19.5973 (4) Å
*V* = 1957.43 (6) Å^3^
*Z* = 4
Cu *K*α radiation
μ = 3.19 mm^−1^
*T* = 100 K
0.16 × 0.06 × 0.05 mm

#### Data collection

Bruker APEX DUO 4K CCD diffractometer
Absorption correction: multi-scan (*SADABS*; Bruker, 2008 ▶) *T*_min_ = 0.630, *T*_max_ = 0.857
14434 measured reflections
3241 independent reflections
3108 reflections with *I* > 2σ(*I*)
*R*_int_ = 0.038

#### Refinement

*R*[*F* ^2^ > 2σ(*F* ^2^)] = 0.023
*wR*(*F* ^2^) = 0.055
*S* = 1.05
3241 reflections
245 parameters
H-atom parameters constrained
Δρ_max_ = 0.14 e Å^−3^
Δρ_min_ = −0.19 e Å^−3^
Absolute structure: Flack (1983 ▶), 1327 Friedel Pairs
Flack parameter: 0.009 (9)

### 

Data collection: *APEX2* (Bruker, 2011 ▶); cell refinement: *SAINT* (Bruker, 2008 ▶); data reduction: *SAINT* and *XPREP* (Bruker, 2008 ▶); program(s) used to solve structure: *SIR97* (Altomare *et al.*, 1999 ▶); program(s) used to refine structure: *SHELXL97* (Sheldrick, 2008 ▶); molecular graphics: *DIAMOND* (Brandenburg & Putz, 2005 ▶); software used to prepare material for publication: *WinGX* (Farrugia, 1999 ▶).

## Supplementary Material

Crystal structure: contains datablock(s) global, I. DOI: 10.1107/S1600536811040815/pv2453sup1.cif

Structure factors: contains datablock(s) I. DOI: 10.1107/S1600536811040815/pv2453Isup2.hkl

Additional supplementary materials:  crystallographic information; 3D view; checkCIF report

## Figures and Tables

**Table 1:** Hydrogen-bond geometry (Å, °) *Cg*1 and *Cg*2 are the centroids of the C1–C7 and C17–C22 rings, respectively.

*D*—H⋯*A*	*D*—H	H⋯*A*	*D*⋯*A*	*D*—H⋯*A*
O3—H3*A*⋯O3^i^	0.84	2.18	2.9905 (10)	163
C13—H13⋯O2^ii^	1.00	2.45	3.4005 (19)	158
C18—H18⋯O3^i^	0.95	2.58	3.521 (2)	171
C20—H20⋯Cl1^iii^	0.95	2.75	3.6268 (19)	154
C8—H8*B*⋯*Cg*1^iv^	0.99	2.76	3.7480 (18)	175
C16—H16*B*⋯*Cg*2^iv^	0.99	2.79	3.7195 (17)	156

Symmetry codes: (i) 
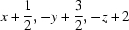
; (ii) 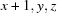; (iii) 
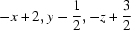
; (iv) 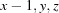.

**Fig. 1.**
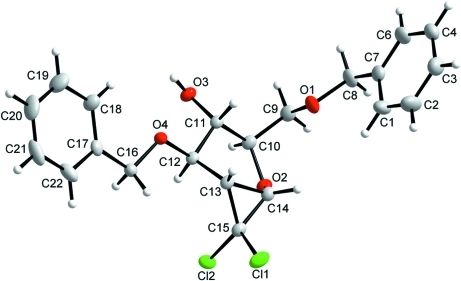
A view of the title compound; displacement ellipsoids are drawn at 50% probability level.

**Fig. 2.**
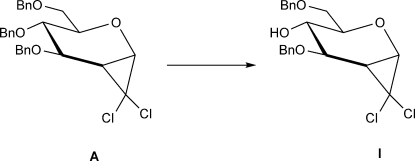
Reaction scheme for the dihalocarbene cyclopropanation of the protected glucal.

## supplementary crystallographic information

### Comment

A combination of AlCl_3_ and a base additive such as *N*,*N*-dimethylaniline cleaves benzyl ethers efficiently (Akiyama *et al.*, 1991). We have found that treatment of dichlorocyclopropyl sugar derivative A with AlCl_3_ in toluene and in the absence of a base additive resulted to selective deprotection and afforded 4-*O*-debenzylated sugar derivative I in 73% yield (Fig. 2). Although ^1^H NMR data could be used to establish the structure of the product, the highly reactive and strained bicyclic junction requires single-crystal X-ray diffraction study to determine the stereochemistry of the product (Shanmugasundaram *et al.*, 2002)

In the title compound (Fig. 1) the *O-*-benzyl groups are all in equatorial positions. The pyran ring adopts a twist-boat conformation with ring puckering parameters of q_2_ = 0.6935 (15) Å, q_3_ = -0.1106 (16) Å, Q = 0.7023 (16) Å and φ_2_ = 342.07 (14)° (Cremer & Pople, 1975). Strong O—H···O hydrogen bonding create infinite one-dimensional chains along the [100] direction. Several weak C—H···O/Cl/*Cg* interactions are also noted and listed in Table 1.

### Experimental

AlCl_3_ (37 mg, 0.28 mmol) was added to a solution of dichlorocyclopropyl sugar derivative (see A in scheme 2)(100 mg, 0.20 mmol) in toluene (1 ml) and the resulting mixture was stirred for 2 h at room temperature. The reaction mixture was then diluted with water and the aqueous phase was extracted with toluene. The combined organic phases were dried over MgSO_4_, filtered and evaporated *in vacuo*. Chromatography on silica gel (ethyl acetate/hexane, 5:95) of the residue and recrystallization from hexane gave dichlorocyclopropyl sugar derivative I in 73% yield as a white solid.

### Refinement

All hydrogen atoms were positioned in geometrically idealized positions with C—H = 1.00, 0.99, 0.95 and 0.84 Å for methine, methylene, aromatic and hydroxyl H atoms respectively. All hydrogen atoms were allowed to ride on their parent atoms with *U*_iso_(H) = 1.2*U*_eq_, except for the hydroxyl where *U*_iso_(H) = 1.5*U*_eq_ was utilized. The initial positions of hydroxyl hydrogen atom was located from a Fourier difference map and refined as fixed rotor. The D enantiomer refined to a final Flack parameter of 0.009 (9).

### Crystal data

**Table:**

C_21_H_22_Cl_2_O_4_	*F*(000) = 856
*M**_r_* = 409.29	*D*_x_ = 1.389 Mg m^−^^3^
Orthorhombic, *P*2_1_2_1_2_1_	Cu *K*α radiation, λ = 1.54178 Å
Hall symbol: P 2ac 2ab	Cell parameters from 8719 reflections
*a* = 5.2985 (1) Å	θ = 4.5–64.5°
*b* = 18.8511 (3) Å	µ = 3.19 mm^−^^1^
*c* = 19.5973 (4) Å	*T* = 100 K
*V* = 1957.43 (6) Å^3^	Needle, colorless
*Z* = 4	0.16 × 0.06 × 0.05 mm

### Data collection

**Table:**

Bruker APEX DUO 4K CCD diffractometer	3241 independent reflections
Incoatec Quazar Multilayer Mirror	3108 reflections with *I* > 2σ(*I*)
Detector resolution: 8.4 pixels mm^-1^	*R*_int_ = 0.038
φ and ω scans	θ_max_ = 64.9°, θ_min_ = 4.5°
Absorption correction: multi-scan (*SADABS*; Bruker, 2008)	*h* = −3→6
*T*_min_ = 0.630, *T*_max_ = 0.857	*k* = −21→22
14434 measured reflections	*l* = −22→22

### Refinement

**Table:**

Refinement on *F*^2^	Secondary atom site location: difference Fourier map
Least-squares matrix: full	Hydrogen site location: inferred from neighbouring sites
*R*[*F*^2^ > 2σ(*F*^2^)] = 0.023	H-atom parameters constrained
*wR*(*F*^2^) = 0.055	*w* = 1/[σ^2^(*F*_o_^2^) + (0.0249*P*)^2^ + 0.1406*P*] where *P* = (*F*_o_^2^ + 2*F*_c_^2^)/3
*S* = 1.05	(Δ/σ)_max_ = 0.001
3241 reflections	Δρ_max_ = 0.14 e Å^−^^3^
245 parameters	Δρ_min_ = −0.19 e Å^−^^3^
0 restraints	Absolute structure: Flack (1983), 1327 Friedel Pairs
Primary atom site location: structure-invariant direct methods	Flack parameter: 0.009 (9)

### Special details

**Table:**

Experimental. The intensity data was collected on a Bruker Apex DUO 4 K CCD diffractometer using an exposure time of 10 s/frame. A total of 1989 frames were collected with a frame width of 1° covering up to θ = 64.94° with 97.8% completeness accomplished.Analytical data: mp 91–93 °C; ^1^H NMR (CDCl_3_, 400 MHz) δ 7.50–7.20 (m, 10H), 4.77 (d, *J* = 11.7 Hz, 1H), 4.61–4.50 (m, 3H), 3.90–3.80 (m, 4H), 3.65 (dd, *J* = 1.5 and 3.3 Hz, 1H), 3.56 (dd, *J* = 4.4 and 9.2 Hz, 1H), 2.59 (bs, 1H), 1.73 (dd, *J* = 4.6 and 8.2 Hz, 1H); ^13^C NMR (CDCl_3_, 75 MHz) δ 137.9, 137.1, 128.6, 128.4, 128.2, 127.7, 127.6, 79.2, 76.4, 73.5, 71.9, 70.5, 68.3, 58.9, 33.2.
Geometry. All e.s.d.'s (except the e.s.d. in the dihedral angle between two l.s. planes) are estimated using the full covariance matrix. The cell e.s.d.'s are taken into account individually in the estimation of e.s.d.'s in distances, angles and torsion angles; correlations between e.s.d.'s in cell parameters are only used when they are defined by crystal symmetry. An approximate (isotropic) treatment of cell e.s.d.'s is used for estimating e.s.d.'s involving l.s. planes.
Refinement. Refinement of *F*^2^ against ALL reflections. The weighted *R*-factor *wR* and goodness of fit *S* are based on *F*^2^, conventional *R*-factors *R* are based on *F*, with *F* set to zero for negative *F*^2^. The threshold expression of *F*^2^ > σ(*F*^2^) is used only for calculating *R*-factors(gt) *etc*. and is not relevant to the choice of reflections for refinement. *R*-factors based on *F*^2^ are statistically about twice as large as those based on *F*, and *R*- factors based on ALL data will be even larger.

### Fractional atomic coordinates and isotropic or equivalent isotropic displacement parameters (Å^2^)

**Table:**

	*x*	*y*	*z*	*U*_iso_*/*U*_eq_	
C1	0.5355 (4)	1.08839 (10)	0.90087 (9)	0.0335 (4)	
H1	0.4672	1.0591	0.866	0.04*	
C2	0.7181 (4)	1.13816 (11)	0.88478 (10)	0.0425 (5)	
H2	0.7732	1.143	0.8389	0.051*	
C3	0.8206 (4)	1.18073 (11)	0.93482 (10)	0.0354 (5)	
H3	0.9442	1.2153	0.9234	0.042*	
C4	0.7428 (3)	1.17297 (9)	1.00165 (9)	0.0292 (4)	
H4	0.8145	1.2017	1.0365	0.035*	
C6	0.5596 (3)	1.12310 (9)	1.01784 (9)	0.0287 (4)	
H6	0.5073	1.1177	1.0639	0.034*	
C7	0.4516 (3)	1.08095 (9)	0.96753 (9)	0.0242 (4)	
C8	0.2384 (4)	1.03164 (9)	0.98508 (9)	0.0277 (4)	
H8A	0.2523	1.017	1.0335	0.033*	
H8B	0.0754	1.0565	0.979	0.033*	
C9	0.0295 (3)	0.92729 (9)	0.95300 (8)	0.0228 (4)	
H9A	−0.1252	0.9554	0.9444	0.027*	
H9B	0.0258	0.9108	1.0009	0.027*	
C10	0.0368 (3)	0.86420 (8)	0.90551 (8)	0.0190 (3)	
H10	−0.1134	0.8344	0.917	0.023*	
C11	0.2697 (3)	0.81673 (8)	0.91569 (8)	0.0169 (3)	
H11	0.417	0.8468	0.9292	0.02*	
C12	0.3303 (3)	0.77835 (8)	0.84966 (8)	0.0165 (3)	
H12	0.1789	0.7518	0.8331	0.02*	
C13	0.4032 (3)	0.83516 (8)	0.79898 (8)	0.0175 (3)	
H13	0.5868	0.8472	0.7966	0.021*	
C14	0.2225 (3)	0.89727 (8)	0.79761 (8)	0.0193 (3)	
H14	0.2957	0.9461	0.7955	0.023*	
C15	0.2516 (3)	0.85065 (9)	0.73630 (8)	0.0198 (3)	
C16	0.5667 (3)	0.68293 (8)	0.80641 (8)	0.0190 (3)	
H16A	0.6067	0.7097	0.7643	0.023*	
H16B	0.4088	0.6561	0.7985	0.023*	
C17	0.7775 (3)	0.63247 (8)	0.82228 (8)	0.0192 (3)	
C18	0.8761 (3)	0.62666 (9)	0.88720 (9)	0.0258 (4)	
H18	0.8135	0.6562	0.9227	0.031*	
C19	1.0653 (4)	0.57824 (11)	0.90107 (11)	0.0381 (5)	
H19	1.1307	0.5746	0.9461	0.046*	
C20	1.1593 (3)	0.53552 (10)	0.85055 (12)	0.0404 (5)	
H20	1.2892	0.5024	0.8605	0.048*	
C21	1.0652 (3)	0.54082 (10)	0.78545 (12)	0.0401 (5)	
H21	1.1301	0.5113	0.7503	0.048*	
C22	0.8757 (3)	0.58918 (10)	0.77109 (10)	0.0319 (4)	
H22	0.812	0.5929	0.7259	0.038*	
O1	0.2452 (2)	0.97025 (6)	0.94223 (6)	0.0233 (3)	
O2	0.00229 (19)	0.88752 (6)	0.83550 (5)	0.0204 (2)	
O3	0.2084 (2)	0.76993 (6)	0.97025 (5)	0.0212 (3)	
H3A	0.3402	0.7498	0.9841	0.032*	
O4	0.5327 (2)	0.73099 (5)	0.86182 (5)	0.0177 (2)	
Cl1	0.41378 (7)	0.88614 (2)	0.66614 (2)	0.02898 (11)	
Cl2	0.00637 (7)	0.79466 (2)	0.711124 (19)	0.02339 (10)	

### Atomic displacement parameters (Å^2^)

**Table:**

	*U*^11^	*U*^22^	*U*^33^	*U*^12^	*U*^13^	*U*^23^
C1	0.0449 (12)	0.0313 (10)	0.0242 (9)	−0.0091 (9)	−0.0094 (8)	−0.0001 (8)
C2	0.0609 (14)	0.0429 (12)	0.0238 (10)	−0.0156 (11)	−0.0032 (10)	0.0071 (9)
C3	0.0414 (11)	0.0277 (10)	0.0370 (11)	−0.0071 (9)	−0.0040 (8)	0.0040 (9)
C4	0.0328 (11)	0.0236 (9)	0.0312 (10)	0.0032 (8)	−0.0041 (8)	−0.0067 (8)
C6	0.0321 (10)	0.0271 (10)	0.0270 (9)	0.0047 (8)	0.0042 (7)	−0.0073 (8)
C7	0.0249 (10)	0.0191 (9)	0.0286 (9)	0.0066 (7)	−0.0018 (7)	−0.0017 (7)
C8	0.0302 (10)	0.0220 (9)	0.0309 (10)	0.0035 (8)	0.0049 (8)	−0.0091 (8)
C9	0.0180 (9)	0.0255 (9)	0.0248 (9)	0.0044 (7)	0.0039 (7)	0.0002 (7)
C10	0.0153 (8)	0.0219 (8)	0.0197 (8)	0.0009 (7)	0.0019 (6)	0.0030 (6)
C11	0.0134 (8)	0.0182 (8)	0.0192 (8)	−0.0015 (7)	−0.0013 (6)	0.0021 (6)
C12	0.0118 (8)	0.0184 (8)	0.0193 (8)	0.0006 (6)	−0.0017 (6)	0.0011 (7)
C13	0.0126 (7)	0.0194 (8)	0.0206 (8)	−0.0007 (6)	0.0001 (6)	0.0026 (7)
C14	0.0159 (8)	0.0188 (8)	0.0231 (8)	−0.0007 (6)	−0.0010 (6)	0.0025 (7)
C15	0.0165 (8)	0.0216 (9)	0.0212 (8)	−0.0017 (7)	−0.0001 (6)	0.0047 (7)
C16	0.0207 (8)	0.0171 (8)	0.0192 (8)	−0.0007 (6)	−0.0016 (6)	−0.0012 (6)
C17	0.0155 (8)	0.0145 (8)	0.0277 (9)	−0.0041 (6)	0.0024 (7)	−0.0010 (7)
C18	0.0231 (9)	0.0281 (10)	0.0261 (9)	0.0049 (8)	0.0015 (7)	0.0037 (8)
C19	0.0298 (11)	0.0417 (12)	0.0429 (12)	0.0095 (9)	−0.0045 (8)	0.0103 (10)
C20	0.0234 (10)	0.0260 (10)	0.0717 (16)	0.0089 (8)	−0.0016 (9)	0.0021 (11)
C21	0.0231 (10)	0.0260 (10)	0.0712 (15)	0.0002 (8)	0.0070 (10)	−0.0215 (10)
C22	0.0234 (9)	0.0328 (10)	0.0395 (11)	−0.0008 (8)	−0.0024 (8)	−0.0179 (8)
O1	0.0220 (6)	0.0199 (6)	0.0280 (6)	0.0017 (5)	0.0058 (5)	−0.0077 (5)
O2	0.0156 (5)	0.0245 (6)	0.0210 (5)	0.0039 (5)	−0.0006 (4)	0.0016 (5)
O3	0.0197 (6)	0.0251 (6)	0.0188 (6)	0.0016 (5)	0.0013 (5)	0.0058 (5)
O4	0.0183 (6)	0.0179 (5)	0.0170 (5)	0.0036 (5)	−0.0025 (4)	−0.0011 (4)
Cl1	0.0229 (2)	0.0396 (3)	0.0245 (2)	−0.00114 (19)	0.00327 (16)	0.0137 (2)
Cl2	0.01837 (19)	0.0297 (2)	0.02214 (19)	−0.00268 (17)	−0.00245 (15)	−0.00181 (16)

### Geometric parameters (Å, °)

**Table:**

C1—C2	1.384 (3)	C12—C13	1.511 (2)
C1—C7	1.387 (3)	C12—H12	1
C1—H1	0.95	C13—C15	1.496 (2)
C2—C3	1.379 (3)	C13—C14	1.513 (2)
C2—H2	0.95	C13—H13	1
C3—C4	1.381 (3)	C14—O2	1.3950 (19)
C3—H3	0.95	C14—C15	1.497 (2)
C4—C6	1.388 (3)	C14—H14	1
C4—H4	0.95	C15—Cl2	1.7452 (16)
C6—C7	1.389 (2)	C15—Cl1	1.7541 (16)
C6—H6	0.95	C16—O4	1.4254 (18)
C7—C8	1.503 (3)	C16—C17	1.500 (2)
C8—O1	1.4303 (19)	C16—H16A	0.99
C8—H8A	0.99	C16—H16B	0.99
C8—H8B	0.99	C17—C18	1.380 (2)
C9—O1	1.416 (2)	C17—C22	1.394 (2)
C9—C10	1.511 (2)	C18—C19	1.383 (2)
C9—H9A	0.99	C18—H18	0.95
C9—H9B	0.99	C19—C20	1.370 (3)
C10—O2	1.4523 (18)	C19—H19	0.95
C10—C11	1.538 (2)	C20—C21	1.373 (3)
C10—H10	1	C20—H20	0.95
C11—O3	1.4239 (18)	C21—C22	1.385 (3)
C11—C12	1.517 (2)	C21—H21	0.95
C11—H11	1	C22—H22	0.95
C12—O4	1.4157 (18)	O3—H3A	0.84
			
C2—C1—C7	120.47 (17)	C11—C12—H12	110.3
C2—C1—H1	119.8	C15—C13—C12	122.73 (13)
C7—C1—H1	119.8	C15—C13—C14	59.65 (10)
C3—C2—C1	120.52 (19)	C12—C13—C14	113.49 (13)
C3—C2—H2	119.7	C15—C13—H13	116.1
C1—C2—H2	119.7	C12—C13—H13	116.1
C2—C3—C4	119.70 (19)	C14—C13—H13	116.1
C2—C3—H3	120.2	O2—C14—C15	115.86 (13)
C4—C3—H3	120.2	O2—C14—C13	114.71 (13)
C3—C4—C6	119.82 (17)	C15—C14—C13	59.64 (10)
C3—C4—H4	120.1	O2—C14—H14	117.9
C6—C4—H4	120.1	C15—C14—H14	117.9
C4—C6—C7	120.86 (16)	C13—C14—H14	117.9
C4—C6—H6	119.6	C13—C15—C14	60.72 (10)
C7—C6—H6	119.6	C13—C15—Cl2	120.91 (11)
C1—C7—C6	118.60 (17)	C14—C15—Cl2	120.36 (11)
C1—C7—C8	121.26 (16)	C13—C15—Cl1	117.06 (11)
C6—C7—C8	120.05 (16)	C14—C15—Cl1	117.12 (11)
O1—C8—C7	110.31 (13)	Cl2—C15—Cl1	111.95 (9)
O1—C8—H8A	109.6	O4—C16—C17	109.83 (12)
C7—C8—H8A	109.6	O4—C16—H16A	109.7
O1—C8—H8B	109.6	C17—C16—H16A	109.7
C7—C8—H8B	109.6	O4—C16—H16B	109.7
H8A—C8—H8B	108.1	C17—C16—H16B	109.7
O1—C9—C10	109.73 (12)	H16A—C16—H16B	108.2
O1—C9—H9A	109.7	C18—C17—C22	118.41 (16)
C10—C9—H9A	109.7	C18—C17—C16	121.58 (14)
O1—C9—H9B	109.7	C22—C17—C16	120.01 (15)
C10—C9—H9B	109.7	C17—C18—C19	120.55 (17)
H9A—C9—H9B	108.2	C17—C18—H18	119.7
O2—C10—C9	109.91 (12)	C19—C18—H18	119.7
O2—C10—C11	113.57 (12)	C20—C19—C18	120.64 (19)
C9—C10—C11	113.50 (13)	C20—C19—H19	119.7
O2—C10—H10	106.4	C18—C19—H19	119.7
C9—C10—H10	106.4	C19—C20—C21	119.78 (18)
C11—C10—H10	106.4	C19—C20—H20	120.1
O3—C11—C12	113.16 (12)	C21—C20—H20	120.1
O3—C11—C10	105.95 (12)	C20—C21—C22	119.98 (18)
C12—C11—C10	109.68 (12)	C20—C21—H21	120
O3—C11—H11	109.3	C22—C21—H21	120
C12—C11—H11	109.3	C21—C22—C17	120.65 (18)
C10—C11—H11	109.3	C21—C22—H22	119.7
O4—C12—C13	111.35 (12)	C17—C22—H22	119.7
O4—C12—C11	108.51 (12)	C9—O1—C8	110.77 (12)
C13—C12—C11	106.06 (12)	C14—O2—C10	115.94 (11)
O4—C12—H12	110.3	C11—O3—H3A	109.5
C13—C12—H12	110.3	C12—O4—C16	111.61 (11)
			
C7—C1—C2—C3	0.5 (3)	C12—C13—C15—Cl2	−9.9 (2)
C1—C2—C3—C4	0.9 (3)	C14—C13—C15—Cl2	−109.85 (14)
C2—C3—C4—C6	−0.9 (3)	C12—C13—C15—Cl1	−152.58 (12)
C3—C4—C6—C7	−0.4 (3)	C14—C13—C15—Cl1	107.49 (13)
C2—C1—C7—C6	−1.7 (3)	O2—C14—C15—C13	−104.74 (15)
C2—C1—C7—C8	174.77 (17)	O2—C14—C15—Cl2	6.00 (19)
C4—C6—C7—C1	1.7 (3)	C13—C14—C15—Cl2	110.74 (14)
C4—C6—C7—C8	−174.85 (16)	O2—C14—C15—Cl1	147.88 (11)
C1—C7—C8—O1	34.7 (2)	C13—C14—C15—Cl1	−107.39 (13)
C6—C7—C8—O1	−148.89 (15)	O4—C16—C17—C18	12.7 (2)
O1—C9—C10—O2	−68.30 (16)	O4—C16—C17—C22	−168.31 (14)
O1—C9—C10—C11	60.10 (17)	C22—C17—C18—C19	−0.9 (2)
O2—C10—C11—O3	−149.38 (12)	C16—C17—C18—C19	178.06 (16)
C9—C10—C11—O3	84.13 (15)	C17—C18—C19—C20	0.4 (3)
O2—C10—C11—C12	−26.92 (18)	C18—C19—C20—C21	0.1 (3)
C9—C10—C11—C12	−153.42 (13)	C19—C20—C21—C22	−0.1 (3)
O3—C11—C12—O4	−57.46 (16)	C20—C21—C22—C17	−0.4 (3)
C10—C11—C12—O4	−175.51 (12)	C18—C17—C22—C21	0.9 (3)
O3—C11—C12—C13	−177.19 (12)	C16—C17—C22—C21	−178.06 (16)
C10—C11—C12—C13	64.76 (16)	C10—C9—O1—C8	178.08 (13)
O4—C12—C13—C15	126.95 (15)	C7—C8—O1—C9	−173.42 (14)
C11—C12—C13—C15	−115.19 (16)	C15—C14—O2—C10	117.76 (14)
O4—C12—C13—C14	−165.12 (12)	C13—C14—O2—C10	51.05 (17)
C11—C12—C13—C14	−47.26 (17)	C9—C10—O2—C14	96.56 (15)
C15—C13—C14—O2	106.67 (15)	C11—C10—O2—C14	−31.80 (18)
C12—C13—C14—O2	−8.71 (19)	C13—C12—O4—C16	−75.51 (15)
C12—C13—C14—C15	−115.39 (15)	C11—C12—O4—C16	168.12 (12)
C12—C13—C15—C14	99.94 (16)	C17—C16—O4—C12	−178.33 (12)

### Hydrogen-bond geometry (Å, °)

**Table:**

Cg1 and Cg2 are the centroids of the C1–C7 and C17–C22 rings, respectively.

**Table:**

*D*—H···*A*	*D*—H	H···*A*	*D*···*A*	*D*—H···*A*
O3—H3A···O3^i^	0.84	2.18	2.9905 (10)	163.
C11—H11···O1	1.00	2.51	2.9433 (19)	106.
C12—H12···Cl2	1.00	2.68	3.2266 (15)	114.
C13—H13···O2^ii^	1.00	2.45	3.4005 (19)	158.
C18—H18···O4	0.95	2.37	2.725 (2)	102.
C18—H18···O3^i^	0.95	2.58	3.521 (2)	171.
C20—H20···Cl1^iii^	0.95	2.75	3.6268 (19)	154.
C8—H8B···Cg1^iv^	0.99	2.76	3.7480 (18)	175
C16—H16B···Cg2^iv^	0.99	2.79	3.7195 (17)	156

Symmetry codes: (i) *x*+1/2, −*y*+3/2, −*z*+2; (ii) *x*+1, *y*, *z*; (iii) −*x*+2, *y*−1/2, −*z*+3/2; (iv) *x*−1, *y*, *z*.
